# Changes in the Inflammatory Response to Injury and Its Resolution during the Loss of Regenerative Capacity in Developing *Xenopus* Limbs

**DOI:** 10.1371/journal.pone.0080477

**Published:** 2013-11-20

**Authors:** Anthony L. Mescher, Anton W. Neff, Michael W. King

**Affiliations:** 1 Indiana University Center for Regenerative Biology and Medicine, Indiana University School of Medicine, Bloomington, Indiana, United States of America; 2 Indiana University School of Medicine, Terre Haute, Indiana, United States of America; UNIFESP Federal University of São Paulo, Brazil

## Abstract

Tissue and organ regeneration, unlike development, involves an injury that in postembryonic animals triggers inflammation followed by resolution. How inflammation affects epimorphic regeneration is largely uninvestigated. Here we examine inflammation and its resolution in *Xenopus laevis* hindlimb regeneration, which declines during larval development. During the first 5 days postamputation, both regeneration-competent stage 53 and regeneration-deficient stage 57 hindlimbs showed very rapid accumulation of leukocytes and cells expressing interleukin-1β and matrix metalloproteinase 9. Expression of genes for factors mediating inflammatory resolution appeared more persistent at stages 55 and 57 than at stage 53, suggesting changes in this process during development. *FoxP3*, a marker for regulatory T cells, was upregulated by amputation in limbs at all three stages but only persisted at stage 57, when it was also detected before amputation. Expression of genes for cellular reprogramming, such as *SALL4*, was upregulated in limbs at all 3 stages, but markers of limb patterning, such as *Shh*, were expressed later and less actively after amputation in regeneration-deficient limbs. Topical application of specific proinflammatory agents to freshly amputated limbs increased interleukin-1β expression locally. With aqueous solutions of the proinflammatory metal beryllium sulfate, this effect persisted through 7 days postamputation and was accompanied by inhibition of regeneration. In BeSO_4_-treated limbs expression of markers for both inflammation and resolution, including *FoxP3*, was prolonged, while genes for cellular reprogramming were relatively unaffected and those for limb patterning failed to be expressed normally. These data imply that in *Xenopus* hindlimbs postamputation inflammation and its resolution change during development, with little effect on cellular dedifferentiation or reprogramming, but potentially interfering with the expression of genes required for blastema patterning. The results suggest that developmental changes in the larval anuran immune system may be involved in the ontogenetic loss of epimorphic regeneration in this system.

## Introduction

Best developed among certain teleost fishes and urodele amphibians, the capacity of vertebrates to regenerate appendages (epimorphic regeneration) shows both considerable phylogenetic variation and a general decline during ontogeny [1]. Among anuran amphibians (frogs and toads) regenerative ability in developing larval hindlimbs diminishes gradually and variably, with amputation during or after late premetamorphic stages generally resulting in simple scarring of the stump or an unpatterned regenerate [2], [3]. Tail regeneration in *Xenopus laevis* occurs throughout larval development except during a transient “refractory period” from stages 45 to 47, but also occurs more slowly and imperfectly at late larval stages [4], [5]. The causes of this ontogenic loss of regenerative capacity remain unknown, but we have suggested that that major changes in the premetamorphic anuran immune system [6] may produce local changes in the inflammatory response to injury that interfere with epimorphic regeneration [7].

In *Xenopus* the inflammation triggered by amputation has been shown to involve many local physiological changes, including hypoxia, generation of reactive O_2_ species (ROS), and production of cytokines that recruit and activate neutrophils and monocytes/macrophages [8]–[11]. In mammals sustained signaling by these cells of the innate immune system can activate an adaptive immune response, as dendritic cells undergo maturation/activation and release cytokines that elicit T helper and effector cells [12], [13]. When the inflammation-inducing injury includes grafting genetically disparate cells or stem cells that express new proteins, the adaptive response can result in rejection of the transplanted cells [14].

Inflammation is highly regulated and self-limited, normally leading directly to programmed resolution and a return to local tissue homeostasis with angiogenesis and tissue repair [15]. Formerly considered a largely passive process, resolution involves synthesis and activity of protein and lipid mediators that produce a wide variety of local anti-inflammatory effects including inhibition of both antigen-presenting cell (APC) and T cell function [16]–[18]. The extent of the inflammatory response to injury and the effectiveness of its resolution together determine the eventual outcome of the repair or regenerative process [17], [19].

Monocyte-derived macrophages are of key importance during inflammation and its resolution, with major roles in phagocytosis, antigen-presentation, and production of various cytokines and matrix metalloproteases. Recognition and engulfment of apoptotic cells, mainly leukocytes, by macrophages down-regulates their release of pro-inflammatory mediators and limits fibrosis [20]. The relevance of such effects for epimorphic regeneration is suggested by the recent demonstration that deletion of macrophages in an adult urodele (the axolotl, *Ambystoma mexicanum*) during the initial period after limb amputation resulted in the formation of fibrotic limb stumps and complete blockade of regeneration in all cases [21].

Factors mediating inflammation and resolution are among those most strongly up-regulated by amputation in the *Xenopus* hindlimb transcriptome and proteome [9], [22]. The aim of the present study was to compare various parameters of inflammation and resolution in amputated *Xenopus* hindlimbs at developmental stages capable of essentially complete epimorphic regeneration (stage 53), incompletely patterned regeneration with only 2 or 3 digits (stage 55), and either no regeneration or patterning (stage 57). This experimental design allows tests of the hypothesis that the local response to limb amputation changes during the period when the ability to regenerate normally patterned limbs is gradually lost. The decline in regenerative capacity was found to be accompanied by prolonged expression of several factors mediating resolution of inflammation in the amputated limbs. Treatment of stage 53/54 limb stumps with the persistent proinflammatory agent beryllium blocked regeneration and prolonged expression of markers for both inflammation and resolution. Beryllium treatment had little effect on the up-regulation of a gene for cellular reprogramming, but inhibited expression of several required for limb blastema patterning. The results suggest that the decline in limb regenerative capacity in *Xenopus* is accompanied by ontogenic changes in the inflammatory response to trauma and that the changes primarily impact patterning of a new limb.

## Methods

### Limb Amputation and Blastema Collection

Larval *Xenopus laevis* were raised in the laboratory or obtained commercially (NASCO, Ft. Atkinson, WI.) and hindlimbs were staged according to Nieuwkoop and Faber [23]. Larval axolotls (*A. mexicanum*), 3–4 cm in length, were obtained from the Ambystoma Genetic Stock Center. Hindlimbs at various developmental stages were amputated bilaterally at the mid-zeugopodia. For comparisons of the response to amputation at different developmental stages, tissues from 20 limbs at each stage were collected immediately and at 6 hrs, 1, 3, and 5 days post-amputation tissues from 20 limbs were collected 1 mm proximal to the original plane of amputation and pooled for RNA extraction and gene expression analysis. For comparing the inflammatory responses of intact and isolated limbs, newly amputated hindlimb stumps were treated as described below and immediately explanted to individual cultures, using procedures described previously [24]. At each of the times indicated, 20 explanted limbs and 20 limbs regenerating in vivo were collected and the distal tissues pooled as indicated above. At various stages of regeneration additional limbs were fixed in MEMFA (0.1 M MOPS, 2 mM EGTA, 1 mM MgSO_4_, 3.7% formaldehyde) for histological processing, paraffin sectioning, and H&E staining or for enzyme histochemistry to localize leukocyte myeloperoxidase (MPO; Sigma Aldrich).

The study was conducted in strict accordance the relevant NIH guidelines, with the protocol approved by the Indiana University Bloomington Institutional Animal Care and Use Committee (Protocol Number: 9017). All surgery and animal treatments were performed under benzocaine anesthesia and all efforts were made to minimize suffering.

### Application of Immune Adjuvants

To test whether immunostimulation of the wounded limb tissues affected subsequent events of regeneration newly amputated hindlimb stumps were treated locally with the one of following solutions: polyA-U (Sigma Aldrich), 50 mg/ml in 0.67X phosphate-buffered saline (PBS); Freund’s complete adjuvant (Difco); lipopolysaccharide (LPS), 50 mg/ml mineral oil; BeSO_4_ or NiCl_2_ (various concentrations in 0.67X PBS); mineral oil control. For these applications individual hindlimb stumps of each anesthetized larva were positioned so that solution did not contact the pelvis, tail, or other region and the solution was applied to the cut amputation surface for 30 seconds with a pipette. Immediately after such treatments each larva was rinsed and placed in its tank for observation and subsequent tissue collection. Since amputation wounds were closed by epithelialization within a few hours each immunostimulant was applied topically only one time.

### Reverse-transcription and Quantitative PCR (qPCR)

Analysis of the expression of several genes was carried out using both end-point and quantitative RT-PCR essentially as described [25]. Total RNA samples were extracted using the RNAqueous Micro system (Ambion, USA). Reverse transcription reactions were carried out using 1µg of total RNA purified from indicated sources. Each end-point PCR reaction was carried out using the equivalent of 16.7 ng of input RNA, whereas each qPCR reaction was carried out using the equivalent of 2.8 ng of input RNA. As a control for RNA loading into the RT reaction, expression of *Xenopus laevis* ornithine decarboxylase (ODC) was assayed [25]. Analysis of ODC expression was carried out using 25 cycles, whereas all other genes were analyzed using 30 cycles except for FoxP3 which required 40 cycles (denoted by an asterisk in Figure legend).

Quantitative PCR was performed utilizing the Mx3000P QPCR System (Stratagene, USA.). Fluorescence detection chemistry involved utilization of SYBR green dye master mix (Bio Rad, USA.) and was carried out as described [22]. Each RT reaction was equalized for RNA input by assessing the level of expression of the relatively invariant housekeeping gene ornithine decarboxylase (ODC) and expression of each gene of interest was then normalized to the level of ODC. For determination of expression levels by qPCR, standard curves were run for each gene of interest as well as for the normalizer gene, ODC. Standard curves were performed using purified PCR product for each gene serially diluted over a 625-fold range starting with 100 fg of product. Following normalization of amplification results to ODC, the level of expression of each gene is expressed as a relative ratio to the level present at the time of amputation. Statistical comparisons were made of each gene comparing expression at each time point between untreated limbs and beryllium treated limbs using independent samples T-test.

PCR primer sequences and GenBank accession numbers are:

ODC

Accession #X56316

U 5′-TTGATCATGCACATGTCAAGCCAG-3′

D 5′-ATTGATGCTGGCAGCAGTACAGAC-3′

FGL-2

Accession # BC084819

U 5′-TAAGCTGCTCATCTGAGACACCAG-3′

D 5′-ATAGTGGTACAGCAGCAGGTGATG-3′

FoxP3

Accession # NM_001127727

U 5′-TCACTGGTTCACTAGAATGTTTGC-3′

D 5′-TGATCAATAAGAAAGGACCTGCTC-3′

IL-1β

Accession #AJ010497

U 5′-GGGATGAGGATTTATTAGGCCTTC-3′

D 5′-AAAAGCCATGCTTACCTTCTCCTC-3′

ANXA1

Accession #BC053786

U 5′-CAGATCTGCATTCAGTCCTTGATC-3′

D 5′-GCTTGCCTACGCTCTTTTGGTAAG-3′

SALL1

Accession AF310007

U 5′-AGAACTCTGAACCAAATGCACCTC-3′

D 5′-CAGCTTGTATACACAGCTCTAGTC-3′

SALL4

Accession # AY336983

U 5′-TCACCACATCTCTCCTAAAGCAAG-3′

D 5′-GGTATGAGAACGTAGATGGATTTG-3′

SOCS1

Accession #BE026949

U 5′-GTCGTTAATGCCCTGTGTTAGATC-3′

D 5′-ATGCTGGGAAATTCAGTCTAGATG-3′

SOCS3

Accession #BC054214

U 5′-CTTTAAGGGCCTTGGTTCTGGTTC-3′

D 5′-GCGACGTCACCATTCATTGTCCAG-3′

Shh

Accession #L35248

U 5′-CTATGCAGTCATTGAGGAACACAC-3′

D 5′-CCAAGTCCCTATCTGATACAGTAG-3′

Tbx3

Accession #AB032942

U 5′-ACTAACAAGCCAGTCCTGATGGTC-3′

D 5′-GGAATGTAGTTCAGCAGCAGCTTG-3′

Msx-1

Accession # X58773

U 5′-GAGACCCAAGTGAAGATCTGGTTC-3′

D 5′-ATGGTACATGCTGTATCCAAGGTG-3′

Galectin-Ia

Accession #AB056478

U 5′-GGAAAAATGTCAGCTGGAATGGTC-3′

D 5′-CAAAACAAATCGTTGTCTCCGCTC-3′

XHOXA13

Accession AJ314743

U 5′-GGTGATGTTCCTTTACGACAACAG-3′

D 5′-CAGGGATAATATCCGCTGCCAAAG-3′

## Results and Discussion

### Resolution of inflammation is prolonged after amputation of regeneration-incompetent limbs

**Figure 1:**
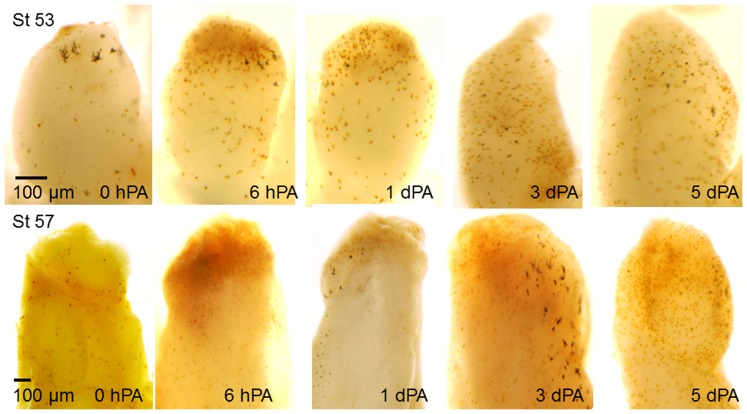
Whole-mounts of stage 53 and stage 57 *Xenopus* hindlimbs from 0 hours to 5 days post-amputation (PA) stained by enzyme histochemistry for myeloperoxidase (MPO), a marker for neutrophils and macrophages. Only background staining is visible at the time of amputation (0 hPA), but by 6 hPA in limbs of both stages the number of brown MPO^+^ cells increase greatly near the site of amputation. MPO staining diminished slowly through 5 dPA in limbs of both stages. The larger, very dark structures are melanocytes.

Developmental stages 53 through 57 cover most of the period during which *Xenopus* hindlimbs lose their capacity for epimorphic regeneration: amputated stage 53 limbs produce well-patterned regenerates typically lacking only the most anterior digit (regeneration-competent), while the larger stage 57 limb stumps are regeneration-deficient, forming at most small, skin-covered cartilaginous spikes. Amputation at either stage elicited a rapid increase in cells staining for myeloperoxidase (MPO), a marker for neutrophils and macrophages, as shown by histochemistry of whole-mounts (Fig. 1). At both stages MPO^+^ cells were abundant in the limb stumps within 6 hours post-amputation (6 hPA), concentrated distally near the cut tissues now covered by wound epithelia. The distal concentration of MPO^+^ cells decreased by one day post-amputation (1 dPA) in limbs of both stages and labeled cells became more dispersed throughout the limb stumps as resolution of the acute inflammatory response began. Three and 5 dPA labeled cells were further dispersed in both stage 53 and stage 57 limb stumps, with more MPO^+^ cells persisting in the older limbs (Fig. 1). These results indicate no obvious difference in amputated limbs at these stages in the accumulation and removal of neutrophils and macrophages.

**Figure 2:**
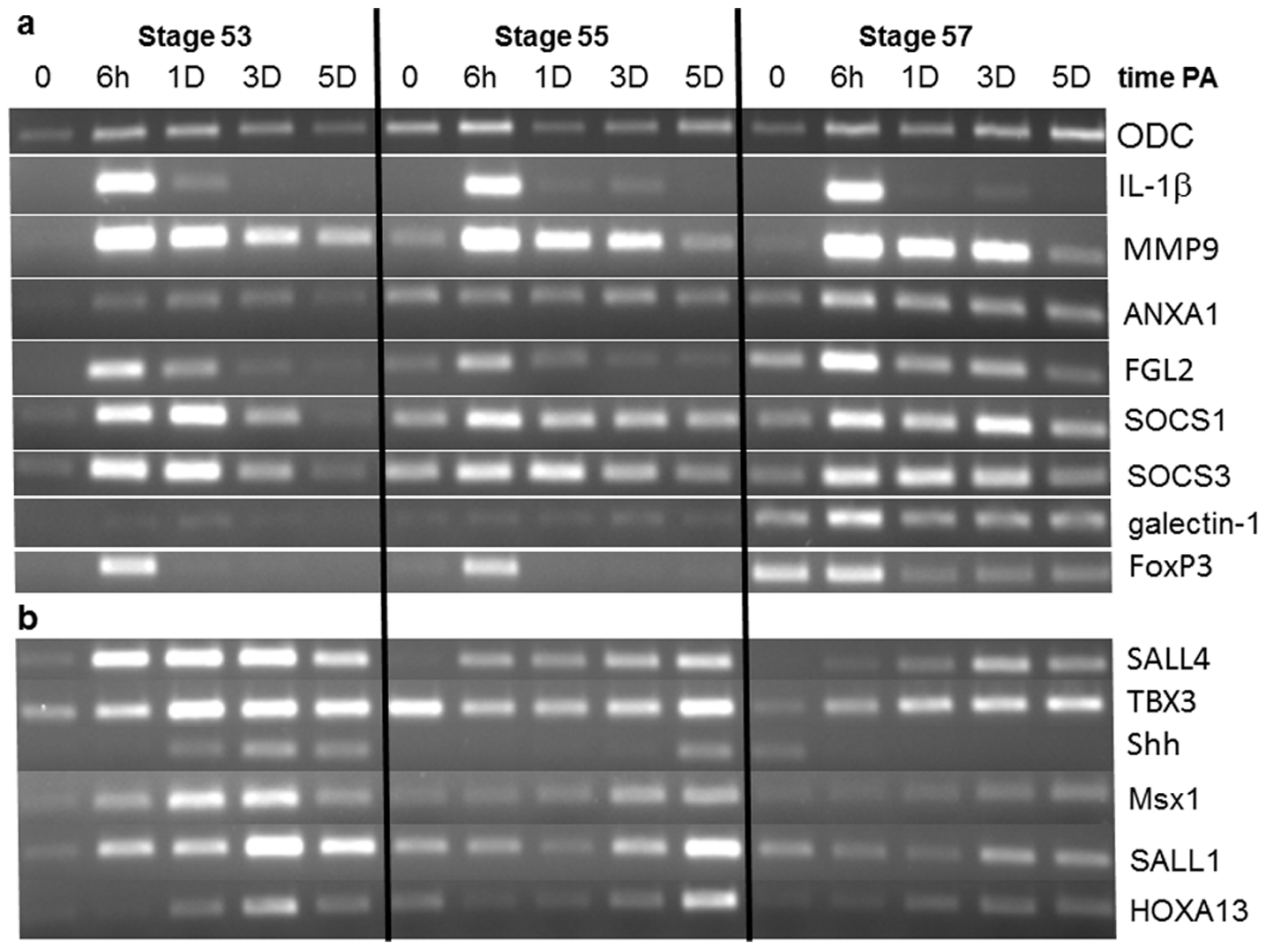
Expression of genes previously identified in studies of gene activity during the early phase of *Xenopus* limb regeneration, shown by RT-PCR at different times PA during the transition from regeneration-complete to –incomplete in stages 53, 55, and 57. Tissue from 20 limb stumps was used for each time point and expression of the loading control ornithine decarboxylase (ODC) is shown in the top row. (a) Genes with primarily inflammation-related function: Expression of interleukin-1β (*IL-1β*) appeared maximal by 6 hPA but then was rapidly diminished at all stages. Expression of factors involved in the resolution of inflammation, including annexin-A1 (ANXA1), fibrinogen-like protein 2 (FGL2), suppressors of cytokine signaling (SOCS) 1 and 3, galectin-1, and the key marker for regulatory T cells, FoxP3, were also all up-regulated by 6hPA and then diminished in limbs of all 3 stages, but more slowly in regeneration-incomplete limbs suggesting that inflammation-related activity persists after amputation in the more developed limbs. Low expression of many of these pro-resolution factors, including FoxP3, was seen already at the time of amputation in regeneration-deficient limbs, indicating the presence of immune cells not found in limbs at early regeneration-complete stages. (b) Genes with primarily cell reprogramming and organ patterning function: Expression of *SALL4* and *TBX3*, both involved in cell reprogramming during early blastema formation, are up-regulated by amputation at all 3 stages, but much more slowly and at apparently lower levels in regeneration-incomplete limbs. Expression of genes required for blastema patterning, including *Shh, Msx1, SALL1,* and *HOXA13*, occurred at various times PA in stage 53 limbs, but was increasingly reduced and delayed in regeneration-deficient limbs, results consistent with the failure of the latter limbs to regenerate with normal patterns. Cycle number for ODC PCR was 25; FoxP3 required 40 cycles and all other genes 30.

Many genes important in inflammation and resolution were identified in our previous microarray and/or proteomic analyses of larval *Xenopus* limb blastemas [9], [22], in addition to genes involved in cellular reprogramming and limb patterning. Expression of several such genes was examined in stage 53, 55 and 57 limbs at various times post-amputation to compare further the local inflammatory response during the larval period when regenerative capacity declines. The key proinflammatory interleukin IL-1β, like the accumulation of MPO^+^ cells, showed strong, transient up-regulation at 6 hPA in limbs at all three stages (Fig. 2a), most likely within macrophages and other antigen-presenting cells [26]. Expression of matrix metalloproteinase-9 (MMP9) also began within hours but continued for at least 3d at all stages. Genes for immunomodulatory proteins involved in resolving inflammation, such as annexin-A1 (*ANXA1* [16], fibrinogen-like protein 2 (*FGL-2*) [27], and suppressors of cytokine signaling (*SOCS1* and *3*) [28], were also expressed more persistently than *IL-1β*. Although up-regulated within 6 hPA at all stages, expression of these resolution factors diminished within 1 day or less at stage 53, but appeared to remain elevated through at least 3 dPA at stage 57 (Fig. 2a). Expression of these factors was also found at low levels in control (unamputated) limbs at stages 55 and 57, but not at stage 53, suggesting differentiation and/or arrival in limbs of cells with immunoregulatory activity during development. Expression of galectin-1, another promoter of resolution [18], was not seen at any time in stage 53 or 55 limbs, but at stage 57 was found in control limbs, with apparent up-regulation by amputation (Fig. 2a).

Galectin-1 is an important product of activated regulatory T cells (T_regs_) [29], which suppress activity of effector T cells during inflammation and have been implicated in the regenerative capacity of tails in *Xenopus* [30]. Expression of the T_reg_ marker FoxP3 occurred transiently at 6 hPA in limbs at all three stages and was detected in stage 57 control limbs but not earlier (Fig. 2a), suggesting that T_regs_ can be elicited by injury as early as stage 53 but are not resident in normal limb tissues until stage 57.

When local expression of genes regulating inflammation was compared with that of genes for cell reprogramming and blastema patterning, the expression profiles were found to be distinctly different (Fig. 2b). Expression of *SALL4*, which characterizes both stem cells and cells undergoing reprogramming [31], [32], began within 1 day and continued through 5 dPA at all three stages of limb development, with weaker and more delayed expression at stage 57 (Fig. 2b). In axolotl limbs *SALL4* is involved in the cellular dedifferentiation that follows amputation, while *SALL1* and *SALL3* are expressed in limb patterning [33]. Previous analyses in *Xenopus* limbs not only showed *SALL4* expression delayed until 3-5 dPA at stage 57, but also found that *SALL4* is not expressed at all in limbs partially transected but not amputated [34].

Expression of genes involved in limb patterning as well as cell reprogramming, such as *TBX3, Shh, Msx1* and *SALL1*, occurred as expected during blastema development in stage 53 limbs but was delayed in stage 55 limbs and was minimal in stage 57 limbs (Fig. 2b). These results suggest that the persistent expression in regeneration-deficient limb stumps of genes involved with inflammation and resolution has little effect on cell reprogramming, but may be incompatible with the normal precisely integrated expression of genes that results in blastema patterning.

Histological observations during the 2 weeks after amputation in regeneration-complete and –deficient hindlimbs were similar to those reported previously [2], [35]. Limbs at regeneration-incompetent stages showed little tissue dedifferentiation, produced only small pseudoblastemas [35], or “fibroblastemas” [36], which during the second week post-amputation formed a layer of dense connective tissue beneath the distal epidermis and in most cases a growing mass of cartilage around the cut, eroding ends of the skeletal elements (data not shown).

### Inflammation following BeSO_4_ treatment reduces regenerative capacity in early larval limb stumps

To test the correlation between prolonged local inflammation and regenerative decline we sought to increase the proinflammatory effect of amputation by applying immune adjuvants to the wound in regeneration-competent hindlimbs (stage 53/54). Poly A-U, LPS, Freund’s complete adjuvant, and NiCl_2_ applied one time to the wound immediately after amputation all caused *IL-1β* expression to persist for at least 1 dPA as measured by RT-PCR, but had no consistent inhibitory effect on limb regeneration (data not shown).

**Figure 3:**
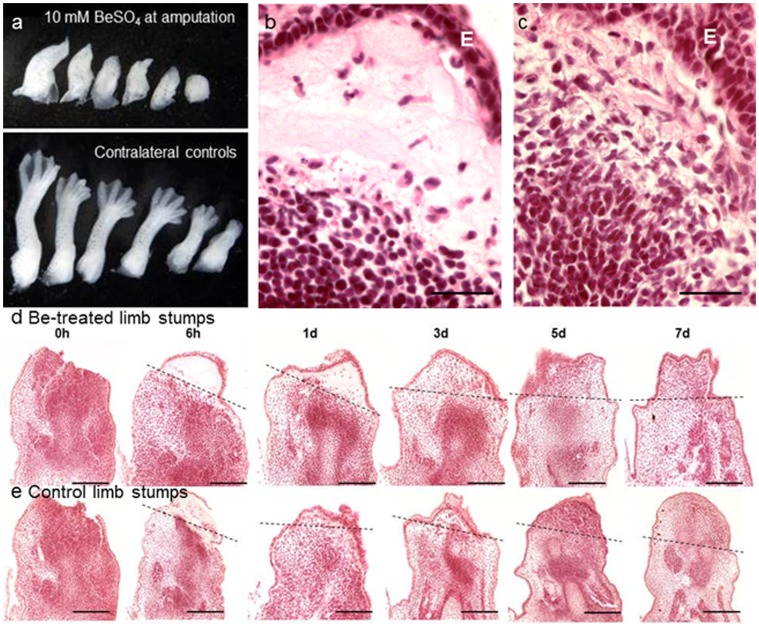
The effect on regeneration of stage 53/54 limbs of local treatment with BeSO_4_ solution (10 mM) immediately after amputation. (a) Epimorphic regeneration was completely inhibited by topical BeSO_4_ while 5 of 6 contralateral limbs regenerated normally. One day PA wound epithelium (E) from adjacent epidermis had migrated across the amputation wound in both Be-treated (b) and control (c) limbs. Treated limbs but not controls also showed subepithelial accumulations of fluid and leukocytes (b). From 6 hours to 7 dPA distal areas of Be-treated limb stumps (d) became less edematous but showed no indication of blastema growth, while controls (e) underwent normal stages of blastema formation and growth. Sectioned tissues stained with hematoxylin & eosin. Bars indicate 50 µm in (b, c) and 100 µm in (d, e). Dashed lines indicate planes of amputation.

However similar treatment with another immunostimulant BeSO_4_ increased edema within the distal limb stump, followed by failure of blastema formation and epimorphic regeneration (Fig. 3). Unlike the other adjuvants tested the beryllium ion persists in exposed tissues and can lead to chronic local inflammation [37]. Be has long been known to inhibit limb regeneration after a very brief exposure in newly amputated larval *Ambystoma* limbs [38]–[40]. Localized exposure of stage 53/54 limb stumps to 10 mM BeSO_4_ increased inflammation at the wound site and completely inhibited regeneration with no mortality (Table 1). Similar treatment with higher concentrations of BeSO_4_ produced more widespread edema and erythema, usually followed by death within about 1 d (Table 1).

**Table 1:** Effects of post-amputation limb treatment with BeSO_4_ in *Xenopus* (stage 53/54).

mM Be n limbs	% mortality	regeneration inflammation				
0		13	0		9/13 (70%)	none
10		10	0		3*/10 (30%)	local
20		14	5/14 (36%)	0		local
40		6	5/6 (83%)	0		systemic
100		5	5/5 (100%)	----		systemic

* all 3 unpatterned “spikes”.

Comparing the responses to Be exposure of *Xenopus* tadpole limbs and larval axolotl limbs of similar size yielded information on the nature of the Be effect (Table 2). The lowest BeSO_4_ concentration (10 mM) that inhibited *Xenopus* regeneration had no apparent effect at all in urodele larvae; there was no sign of local inflammation and all limbs regenerated normally. Only at 40 mM BeSO_4_ did edema and erythema begin to occur locally, with regenerative failure in half the axolotl limbs. At 100 mM BeSO_4_, which was consistently fatal to the anuran larvae, inflammation was increased only locally, while regeneration was blocked completely (Table 2). No axolotls died after localized BeSO_4_ exposure at any concentration. These major differences in mortality and effect on regeneration between *Xenopus* and axolotl larvae after brief reatment of the limbs with BeSO_4_ provided strong initial evidence that the regenerative inhibition is not due to direct toxic effects produced by applying this substance to the cut tissues.

**Table 2:** Effects of post-amputation limb treatment with BeSO_4_ in axolotl larvae (4 cm).

mM Be n limbs	% mortality	regeneration inflammation							
0		16		0		100%			none
10		8		0		100%			none
20		8		0		100%			none
40		14		0		7/14 (50%)		slight, local	
100	8		0		1*/8 (13%)		local		

* spike.

### Inflammation following BeSO_4_ treatment inhibits gene expression needed for patterning and growth of a limb regeneration blastema

**Figure 4:**
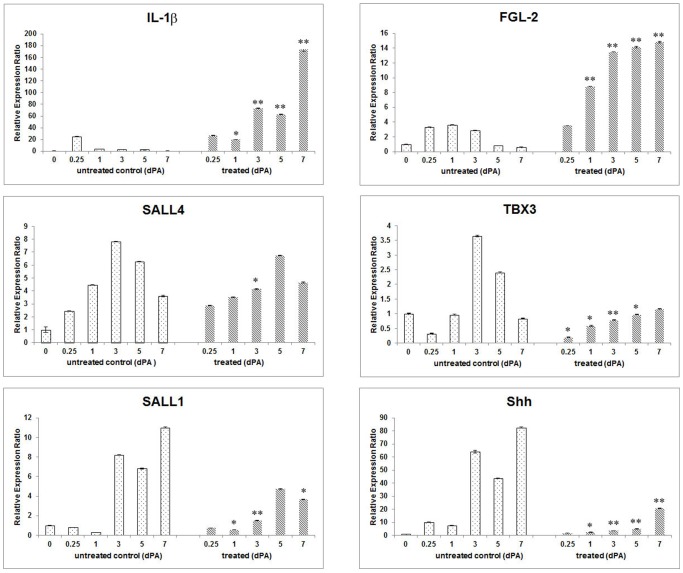
Quantitative PCR of gene expression through 7 days post-amputation in control and 10 mM BeSO_4_-treated stage 53/54 limbs. Tissue from 20 limb stumps was used for each time point. (Top) In untreated control limbs expression of inflammation-related *IL-1β* and *FGL-2* are transient, expression of the patterning genes *Shh* and *SALL1* begins by 3 dPA, and expression of the reprogramming gene *SALL4* occurs throughout this period. In Be-treated limb stumps, *IL-1β* expression persists through 7 dPA, expression of the proresolution factor *FGL-2* is five-fold higher than in controls and also highly persistent, while *SALL4* and *TBX3* expression is similar to that of control regenerates and expression of *SALL1* and *Shh* remains very low. (Bottom) Compared individually expression of proinflammatory *IL-1β* and proresolution *FGL-2* are both significantly elevated and persistent through 7 dPA in Be-treated limbs. In general Be had no significant effect on expression of *SALL4*, but inhibited expression of the patterning-related genes*TBX3, SALL1* and *Shh*. Each time point shows the mean of triplicate PCR runs, with standard deviations. Statistical comparisons are between similar time points in the treated and untreated groups: *  =  P<0.05, **  =  P<0.001, and no symbol indicating no statistical differences.

The inhibition of regeneration by localized immunostimulation at the amputation site was investigated further by quantifying the Be effect on expression of specific genes related to inflammation, cell reprogramming, and blastema patterning. As shown in Figure 4 application of BeSO_4_ at 10 mM to freshly amputated, regeneration-competent limbs stimulated significantly higher and prolonged expression of *IL-1β* and *FGL-2*, which promote and modulate inflammation and resolution but are normally expressed only transiently after amputation at this stage. While enhancing expression of those immunomodulatory genes, Be had no effect on the up-regulation or expression levels of *SALL4* (Fig. 4). The stimulated or normal expression of these genes is additional evidence against a toxic effect of the brief exposure of the limbs to BeSO_4_ at the dose used here.

Unlike these genes involved in either inflammation or cell reprogramming, expression of the patterning-related genes *TBX3*, *Shh* and *SALL1* was significantly inhibited after Be treatment. Although these genes are all strongly up-regulated at 3 dPA in control regeneration-competent limbs, expression of each was significantly reduced in Be-treated limbs. At 7 dPA expression of *Shh* and *SALL1* was only one-third the levels seen in controls (Fig. 4). These results suggest that while cell reprogramming in the limb normally accompanies the inflammation and resolution elicited by amputation, prolonged inflammation disrupts the normal schedule of patterning gene expression. Tissue injury and inflammation have been shown to cause epigenetic changes in local cells that facilitate nuclear reprogramming [41], [42]. Conversely, the microenvironment produced by unresolved inflammation can disrupt tissue patterning by deregulating Wnt and Shh signaling pathways and the dynamic expression of matricellular proteins [43], [44].

**Figure 5:**
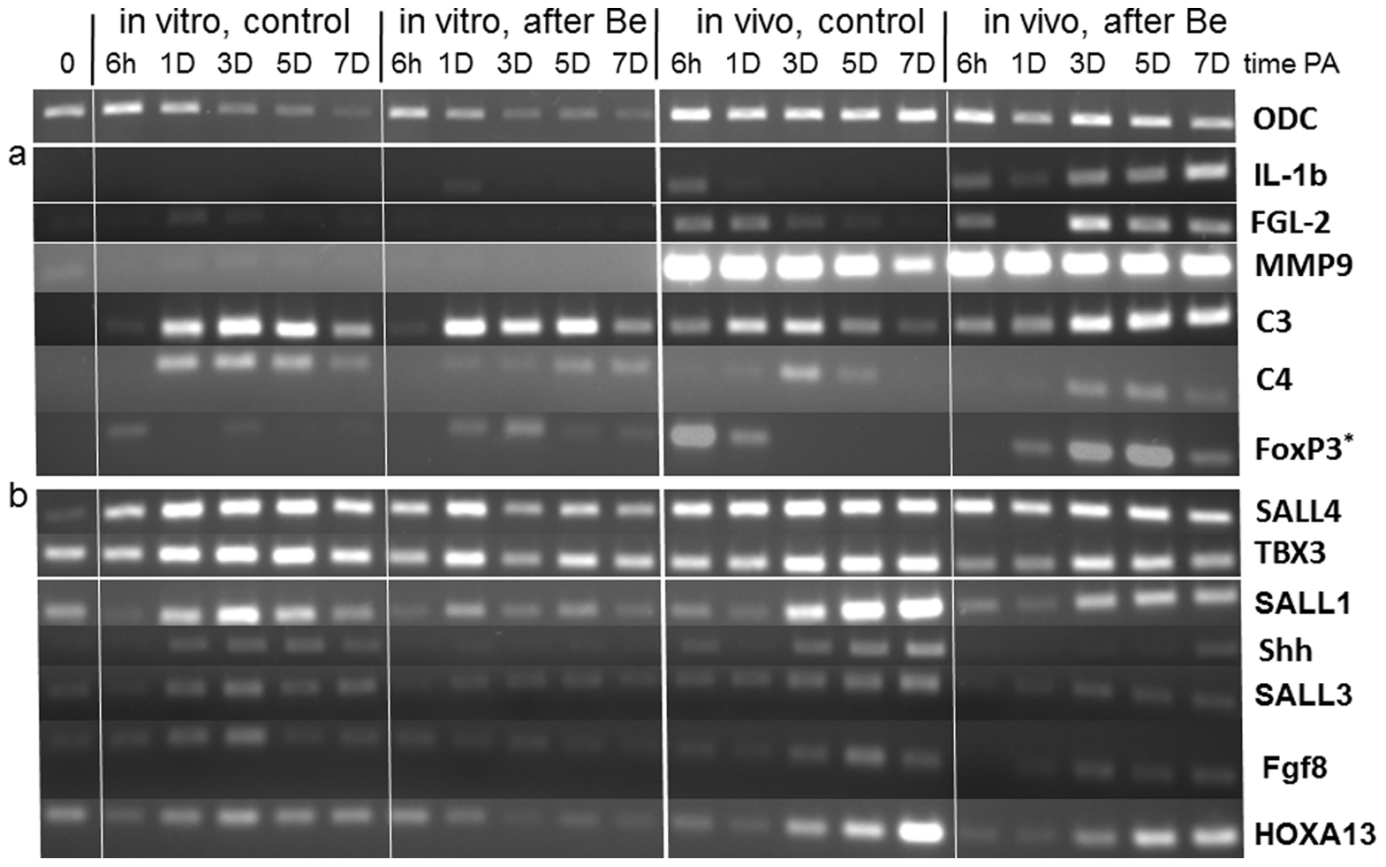
Expression of genes in control and 10_4_-treated stage 53 limbs in vivo and in organ culture at various times after amputation. Although ornithine decarboxylase (ODC) expression (top) is reduced uniformly by explantation, possibly due to decreased cell viability, clear differences in expression of other genes are observed between limb stumps in vivo and in vitro. (a) Expression of the inflammation-related genes *IL-1β, FGL-2, MMP-9,* complement *C3* and *C4*, and of *FoxP3^+^* regulatory T cells, all appear to be up-regulated in Be-treated limb stumps compared to untreated controls. For all these genes except C3 and C4 expression was not seen or was reduced (FoxP3) in explanted limb stumps, indicating that in vivo this gene expression occurs in immigrating leukocytes. (b) In contrast, expression of genes for*SALL4* (which was not inhibited by Be treatment) and limb patterning genes was detected in explanted as well as intact limbs. ^*^FoxP3 PCR required 40 cycles; ODC PCR was carried out for 25 cycles and all other genes were assayed with 30 cycles.

To examine the roles of immigrating cells in the local response to amputation and BeSO_4_ treatment, changes in gene expression during 7 dPA were also compared in stage 53 limbs amputated, with or without exposure to Be, and then either maintained in vivo or immediately explanted to organ culture (Fig. 5). This approach indicated clearly that expression of *IL-1β*, *FGL-2*, and *MMP9*, occurred almost exclusively in cells that enter the limb from blood or elsewhere within hours of amputation (Fig. 5a).However local up-regulation and expression of the complement components C3 and C4 by amputation occur similarly in vivo and in cultured limbs (Fig. 5a), consistent with the report of Del Rio-Tsonis et al. [45] that C3 is synthesized by dedifferentiating cells of the urodele limb. The importance of local complement expression for successful regeneration in a wide variety of models was reviewed recently by Mastellos et al. [46]. Some FoxP3-expressing cells are present in stage 53 limbs are seen to show Be stimulation even after explantation to organ culture. FoxP3-expressing cells also appear to enter limbs during inflammation since the marker’s expression is higher in vivo than in culture (Fig. 5a).

As expected expression of the cell reprogramming and patterning genes was seen in both explanted and intact limb stumps, with the brief exposure to BeSO_4_ having an apparent inhibitory effect that was again minimal with *SALL4* (Fig. 5b).

Since expression of *Shh* and other limb patterning genes likely depends on proliferation and blastema growth, potential cytotoxic or other antiproliferative effects of Be require further consideration. How does Be affect cells and tissues? BeSO_4_ is only weakly carcinogenic or mutagenic and is toxic for cultured cells only after prolonged (hours) exposure at millimolar concentrations [47], [48]. In the early studies with Be and amphibian regeneration, its effect was localized to the exposed cells only, since removal of another 0.5 mm of tissue from the Be-treated stump, either immediately or several days later, was followed by normal regeneration [38], [39], [49]. Tsonis et al. [40] reported that immediate treatment of axolotl limbs with BeSO_4_ blocked the transient increase in inositol phosphate production that occurs locally within a minute after amputation, a result consistent with the similar effects of BeSO_4_ and phosphoinositide-3-kinase inhibitors on differential release of cytokines by LPS-stimulated monocytes and dendritic cells [50].

Thornton [39], [51] and Singer [52] also used regenerating limbs of larval *Ambystoma* and adult newts respectively to test for toxicity after infusing millimolar solutions of Be compounds into blastemas. In those studies blastema growth and regeneration continued, but with increased inflammation, fibrosis and severe patterning defects, results consistent with the data reported here. No studies have determined the percentage of Be remaining in exposed limb tissue following a brief exposure to a specific concentration of a Be compound and washing of the amputation surface. However its lack of effect on expression of *SALL4* and other genes, or the up-regulation of *SALL4* at 5 dPA in stage 57 limbs (not shown), suggests further that Be is not directly cytotoxic.

Virtually all of the acute and chronic human health problems caused by exposure to Be are elicited in susceptible individuals by hypersensitivity reactions involving Be-specific CD4^+^ T cells [48], [53]-[55]. The observation that a concentration of BeSO_4_ which completely inhibits limb regeneration and causes high mortality in *Xenopus* larvae has no effect on regeneration or mortality in axolotl larvae of similar age and size (Table 1) is more consistent with an immune or inflammatory activity than with a cytotoxic effect. The relatively deficient status of urodele adaptive immunity compared to that of *Xenopus* has been reviewed by others [6], [56]–[58]. Anti-inflammatory agents have long been investigated for their effects on wound repair and regeneration. Treatment during inflammation with cortisol or similar immunosuppressive glucocorticoids delays tissue repair [59] and limb regeneration in newts [60]. A high-throughput screen of bioactive compounds affecting tail regeneration in zebrafish larvae revealed no stimulatory molecules but several specific inhibitors, most of which were glucocorticoids [61]. This group found that the sensitive window for glucocorticoid exposure was the first 4 hours after amputation, with inhibition of subsequent wound epithelialization, blastema cell proliferation, and expression of *Junbl, Msxe*, and *Dlx5a.* Local accumulation of neutrophils or macrophages was not significantly reduced by beclomethasone and *PU.1* morphant larvae lacking these cells regenerated tails normally [61]. Glucocorticoid has also been shown to inhibit hindlimb regeneration in stage 53 *Xenopus* larvae [7]. Important glucocorticoid-sensitive events in the immediate aftermath of amputation may include generation of ROS, which is maximal within 1 hour of amputation in *Xenopus* tails and is required for successful regeneration [11].

While corticosteroids generally inhibit wound repair and epimorphic regeneration, more specific anti-inflammatory or immunosuppressive agents can improve patterning in various models of incomplete regeneration. Scarring is reduced in murine full-thickness skin wounds by topical application of celecoxib, which inhibits cyclooxygenase-2 (COX-2) and prostaglandin synthesis [62]. Both celecoxib and diclofenac, another specific COX-2 inhibitor, enhanced regeneration in stage 54/55 (but not later stage) *Xenopus* hindlimbs, as shown by improved anterior-posterior patterning and digit formation [7]. Similar results were obtained with celastrol, an IκB kinase (IKK) inhibitor which blocks NF-κB signaling during immune activation [7]. Fukazawa et al. [30] have shown that celastrol and another IKK inhibitor, as well as the clinically important immunosuppressants cyclosporine A and FK506 (tacrolimus), completely restored the capacity for tail regeneration in *Xenopus* during the transient refractory period (stages 45–47).

Together these inhibitor studies suggest an important balance between inflammation and regeneration. Amputation rapidly triggers gene activity for inflammation and its resolution, as well as for cellular stemness/reprogramming, which is followed by growth and limb patterning. Blastema formation can fail if glucocorticoids are present during the early post-amputation period. Patterning and normal regeneration also fail if inflammation is prolonged locally by the persistent adjuvant Be or perhaps by impaired resolution in the absence of macrophage activity [21]. Conversely, in the presence of COX-2 inhibitors or specific immunosuppressants used to block allogeneic graft rejection and treat autoimmune disorders, regeneration of larval *Xenopus* tails and limbs can be improved.

The ameliorative effects of cyclosporine and other agents targeting the adaptive immune system suggest that the loss of patterning during tissue repair may involve T cell-dependent mechanisms [7], [30]. Down-regulation of both APCs and lymphocytes during resolution is produced in part by annexin-A1, FGL-2, and galectin-1 [63]–[65], which together with *SOCS* and the T_reg_ marker *FoxP3* appear here to be expressed more persistently in regeneration-deficient limb stumps. Activities of these resolving factors and T_regs_ in controlling local immune activity and maintaining self-tolerance in mammals are increasingly well-characterized; their expression in amputated larval *Xenopus* limbs suggests the possibility of similar roles in these tissues’ return to homeostasis after injury.

In *Xenopus laevis* the hindlimbs and the adaptive immune system develop simultaneously. From stage 50, soon after limb buds first appear, to stage 58, when the larval thymus attains its maximal size and the hindlimbs are fully formed, the number of lymphocytes in the thymus increases from 3×10^4^ to 1–2×10^6^ [6]. During metamorphosis these lymphocytes are largely replaced by new T cells which undergo thymic selection for tolerance of newly produced antigens unique to the postmetamorphic frog. Mixed lymphocyte reactions (MLR, in vitro assays for the ability of helper T cells to recognize non-self-antigens, proliferate, and generate effector T cells) between larval and adult immune cells can first be detected at the same stage when limb regenerative ability begins to decline [66].

Recent experiments using limb progenitor cells to impart regenerative capacity to postmetamorphic *Xenopus* underline the importance of inflammation and resolution here [67]. Fibrin gel patches containing both dissociated limb bud cells and beads releasing Shh and FGF10 converted spike growth into multidigit limb formation when applied to the amputation surface at froglet ankles or wrists, with the regenerates developing from both donor and host cells. As expected, long-term survival of these cell allografts, as well as intact limb bud grafts, required immunosuppression of the hosts by prior thymectomy (at stage 48/49). Addition of the anti-inflammatory protein thymosin β4 to the fibrin gel reduced apoptosis and doubled the proliferation rate in donor (but not host) cells, resulting in higher quality regenerates having more digits and metacarpal-like structures with ossification and initial joint development [67]. As the authors suggest, these effects of thymosin β4 are likely due to immunosuppressive activity within the graft since functional T cells and chronic allograft rejection still occur after thymectomy as late as stages 48/49. Thymosin β4 is among the many immunomodulatory factors up-regulated locally by amputation in larval *Xenopus* limbs [68].

Urodeles (salamanders, newts) have well-developed innate immunity but are characterized by “immunodeficient” lymphocytes or APCs, with poor MLR, very slow humoral responses, no memory responses, and slow rejection of skin allografts caused by macrophages or NK cells rather than cytotoxic T cells [56], [58], [69], [70]. Limb regeneration often still occurs after metamorphosis in urodeles, although much more slowly and with defective limb patterning [71]. In the normally neotenic axolotl, skin wounds show scar-free healing [72], but after induced metamorphosis this process is slower and less perfect [73]. Adaptive immunity is enhanced in axolotls after induced metamorphosis [74], which may help explain their reduced regenerative patterning.

Is a role for the developing immune system in the loss of regenerative capacity in *Xenopus* limbs consistent with the long-held view that this ability is an intrinsic property of limb cells? Sessions and Bryant [75] reinforced that idea by reciprocally grafting stage 52/53 hindlimb buds to froglet limb stumps and forelimb “blastemas” to hindlimb stumps at the regeneration-competent stage. The limb bud grafts on froglets regenerated normally after distal amputation, while the pseudoblastemas grafted to young larvae did nothing or formed spikes. This result suggested that the froglet host environment is permissive for regeneration in the grafted limb bud and that the ability to regenerate is an intrinsic property of young larval limb cells and is lost during ontogenesis.

Unknown at the time of that experiment but consistent with the ideas discussed here, anuran skin development includes differentiation of cells resembling Langerhans cells and dendritic cells of mammalian skin [76], [77]. These cells form a reticulum in the skin, with processes interdigitating between adjacent keratinocytes [77]–[79]. In developing *Xenopus* hindlimbs such cells first differentiate in the proximal skin, remaining sparse distally through stage 53 and then gradually becoming more abundant in the distal region as the foot develops [79]. If specialized for antigen-representation like their putative counterparts in mammalian skin, activation of these cells by amputation could interfere with formation of a complete new limb, perhaps by sensitizing T cells against factors required for blastema patterning.

The injury-induced inflammatory response, including both its innate and adaptive components, is increasingly recognized as an important area within regenerative biology. The extensive literature on inflammation as a key determinant of scarring or regeneration in mammalian skin and other systems has revealed complex interactions among immune cells, stem cells and cells undergoing reprogramming. Further investigation of inflammation and resolution in the developing *Xenopus* limb and other models of vertebrate regeneration can be expected to shed light on the capacity for epimorphic regeneration and its attenuation or loss during both ontogeny and phylogeny.
