# Correction: Changes in the Inflammatory Response to Injury and Its Resolution during the Loss of Regenerative Capacity in Developing *Xenopus* Limbs

**DOI:** 10.1371/journal.pone.0110063

**Published:** 2014-09-30

**Authors:** The PLOS ONE Staff

A formatting error occurred Table 1 and Table 2 during the typesetting process of this article. The publisher apologizes for this error. Please see the corrected tables here.

**Table 1:** Effects of post-amputation limb treatment with BeSO_4_ in *Xenopus* (stage 53/54).

mM Be	n limbs	% mortality	regeneration	inflammation
0	13	0	9/13 (70%)	none
10	10	0	3*/10 (30%)	local
20	14	5/14 (36%)	0	local
40	6	5/6 (83%)	0	systemic
100	5	5/5 (100%)	----	systemic

* all 3 unpatterned “spikes”.

**Table 2:** Effects of post-amputation limb treatment with BeSO_4_ in axolotl larvae (4 cm).

mM Be	n limbs	% mortality	regeneration	inflammation
0	16	0	100%	none
10	8	0	100%	none
20	8	0	100%	none
40	14	0	7/14 (50%)	slight, local
100	8	0	1*/8 (13%)	local

* spike.

## References

[1] Mescher AL, Neff AW, King MW (2013) Changes in the Inflammatory Response to Injury and Its Resolution during the Loss of Regenerative Capacity in Developing *Xenopus* Limbs. PLoS ONE 8(11): e80477 doi:10.1371/journal.pone.0080477 24278286 10.1371/journal.pone.0080477 PMC3835323

